# Multidisciplinary Kaizen Event to Improve Adherence to a Sepsis Clinical Care Guideline

**DOI:** 10.1097/pq9.0000000000000435

**Published:** 2021-06-23

**Authors:** Kimberly S. Denicolo, Jacqueline B. Corboy, Norma-Jean E. Simon, Kate J. Balsley, Daniel J. Skarzynski, Emily C. Roben, Elizabeth R. Alpern

**Affiliations:** From the * Division of Emergency Medicine, Emergency Department, Ann & Robert H. Lurie Children’s Hospital of Chicago, Chicago, Ill.; † Department of Pediatrics, Northwestern University, Feinberg School of Medicine; ‡ Division of Emergency Medicine, Data Analytics and Reporting, Ann & Robert H. Lurie Children’s Hospital of Chicago, Chicago, Ill.; § Center for Excellence, Ann & Robert H. Lurie Children’s Hospital of Chicago, Chicago, Ill.; ∥ Lurie Children’s Surgical Foundation, Ann & Robert H. Lurie Children’s Hospital of Chicago, Chicago, Ill.

## Abstract

**Introduction:**

Since 2015, the Ann and Robert H. Lurie Children’s Hospital Emergency Department (ED) has improved the recognition and treatment of pediatric sepsis and septic shock. Despite existing clinical care guidelines, the ED had not yet achieved the Surviving Sepsis Campaign timeliness goals for fluid and antibiotic administration.

**Methods:**

The team conducted a multidisciplinary Kaizen event to evaluate clinical workflows and identify opportunities to improve sepsis care adherence. Using rigorous quality improvement methodology, frontline providers mapped workflows to identify barriers and prioritize emerging solutions.

**Results:**

Thirty-seven staff members across 17 disciplines participated. Nurses and physicians identified communication gaps at pathway initiation. Access to supplies, inadequate task delegation, and a lack of urgency for a subset of pathway patients delayed treatment. Prioritized interventions included scripted communication tools, a delineated response plan, and standardized reassessment processes. Revisions to the key driver diagram were made after the improvement event, guiding future plan-do-study-act cycles.

**Conclusions:**

Frontline provider participation in the Kaizen event uncovered barriers to care and identified the root causes of ineffective communication and system process inefficiencies. Engaging key stakeholders from multiple care areas in a candid context was a novel approach to process improvement within our department. The Kaizen methodology is fundamental to developing sustainable quality improvement practices, creating momentum for a continuous improvement culture to engrain quality improvement in practice. The success of Kaizen will shape the format of future ED improvement projects.

## Abstract 2

Supplemental Digital Content is available in the text.

## INTRODUCTION

Approximately, 75,000 children are treated in the US hospitals each year for severe sepsis and septic shock with an associated mortality rate of up to 20%.^1,2^ Improving early detection and timely initiation of treatment for patients presenting with signs of sepsis has become a priority for American hospitals. One approach to enhance the early recognition and treatment of pediatric patients at risk for sepsis is implementing electronic alerts.^1,3–6^

Despite dedicated quality improvement (QI) efforts, the Emergency Department (ED) at the Ann and Robert H. Lurie Children’s Hospital of Chicago had difficulty meeting time-based goals recommended by the Surviving Sepsis Campaign.^7^ These goals suggest intravenous fluid delivery within 20 minutes and antibiotic infusion within 60 minutes for patients with suspected sepsis.^7^ QI efforts related to sepsis care began in 2015 with bi-weekly meetings of an established multidisciplinary ED sepsis team composed of nurses, providers, and leadership. This group implemented an electronic best-practice alert to identify patients at high risk for sepsis and created sepsis-specific clinical pathways and order set bundles. Although these efforts led to improved recognition, the ED did not achieve time-based goals, reporting time to first fluid bolus of 29.7 minutes and intravenous (IV) antibiotic administration of 73.7 minutes on average.

In July 2018, the ED sepsis team planned and implemented a multidisciplinary, rapid improvement event following Kaizen methodology.^8,9^ This was the first large-scale Kaizen event conducted within the ED at our institution. The purpose of the Kaizen event was 2-fold: (1) to assess if a large-scale event, like a Kaizen, was a feasible and meaningful way to engage frontline staff and other key stakeholders in QI, and, (2) to solicit multiple perspectives about system issues, workflow processes, and process gaps related to the care of patients at risk for sepsis. The ultimate aim of the event was to generate potential solutions and interventions for future plan-do-study-act cycles.

## METHODS

**Fig. 1.**
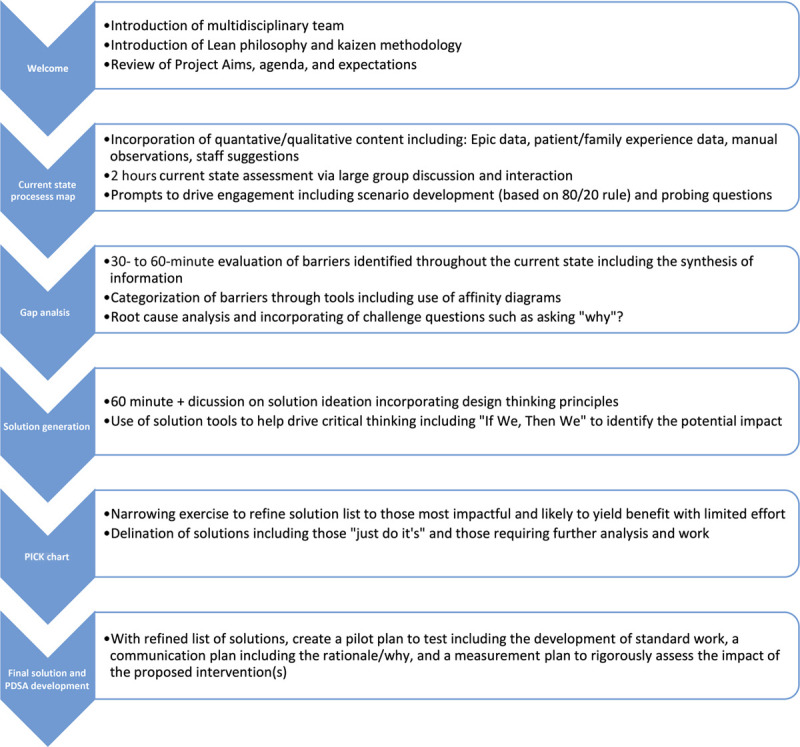
Kaizen facilitator guide.

The Ann and Robert H. Lurie Children’s Hospital of Chicago is an urban, tertiary academic center with an ED patient volume of 56,000 visits annually. In 2015, a vital sign-based electronic sepsis screening tool was implemented, using age-based normative values adapted to our patient population.^10,11^ This tool identifies the earliest point in recognition of a patient with a concern/risk for sepsis, termed “time zero.”^7^ Patients who were identified as “at-risk” by the screening tool were then evaluated (termed “huddle”) by a nurse and provider at the bedside. Following the huddle, the patient is assessed and placed onto our Sepsis pathway (**Figure 1, Supplemental Digital Content 1,** which displays dual pathway, http://links.lww.com/PQ9/A275). The ED sepsis team monitored the electronic screening tool’s implementation monthly, guided by a suite of metrics and standard process improvement charts approved for collection. The project received exempt status from the local Institutional Review Board.

Monthly review of ED sepsis metrics continued to fall short of Surviving Sepsis Campaign goals, prompting the ED sepsis team to investigate trialing a rapid improvement event focused on improving sepsis care and outcomes. “Kaizen,” a Japanese word that translates as “change for the better,” is a methodology that necessitates the engagement of frontline staff in improvement efforts. The Kaizen methodology was introduced in healthcare in the late 1980s. It has increasingly gained traction as a technique to improve healthcare processes. This method’s fundamental philosophy starts with small, easy-to-implement changes and involves soliciting multiple perspectives, allowing multidisciplinary groups to appraise existing clinical workflows critically.^8,12^ The Kaizen approach had not been employed in this pediatric ED previously and was a novel approach for executing process improvement.

The Kaizen’s core planning team included an ED nurse, physician, and operations manager trained in QI methodology. This planning team designed and executed the 1-day rapid improvement event for multidisciplinary frontline staff involved in ED sepsis care. Based on informal clinician feedback of sepsis care concerns, the planning team and nursing colleagues spent 20 hours performing focused chart reviews. These reviews assessed the scope of current practice, revealing trends in treatment that had not conformed to the existing guideline and established the event’s focus. Two patient cases were selected from the chart review to serve as vignettes for process mapping and workflow analysis. Both scenarios involved patients who experienced clinical decompensation, requiring intensive interventions for septic shock management.

The Kaizen was planned during a low patient volume season (summer) and advertised via email and departmental postings three months in advance to allow for adequate clinician representation and appropriate unit staffing. A facilitation guide was developed to focus on probing questions for leading discussions to uncover root-cause barriers (Fig. 1). The planning team identified key stakeholders, contacted area leadership teams with detailed event goals, and requested frontline participation. Leadership engagement was essential to provide approval for frontline clinicians to attend within their regular budgeted working hours. Clinical shifts for those participating were supplemented by other staff members using department budgeted meeting time, allowing for attendance without overtime pay. Physicians participated in using non-clinical salary time. The event’s direct costs, covered by the ED, included event supplies (paper, markers, Post-its) and lunch for all. This event utilized available space within our institution at no cost.

Stakeholders invited to the event included physicians and advanced practice nurses from the ED and hospital medicine services, ED nurses and paramedics, and representatives from the hospital’s Center for Excellence, pharmacy, data analytics, laboratory, registration, and vascular access teams. Essential members of ED leadership, including the ED medical director and senior nursing director, were present. Ground rules set expectations for the group, including an active participation model and respect for individual opinions.

**Fig. 2.**
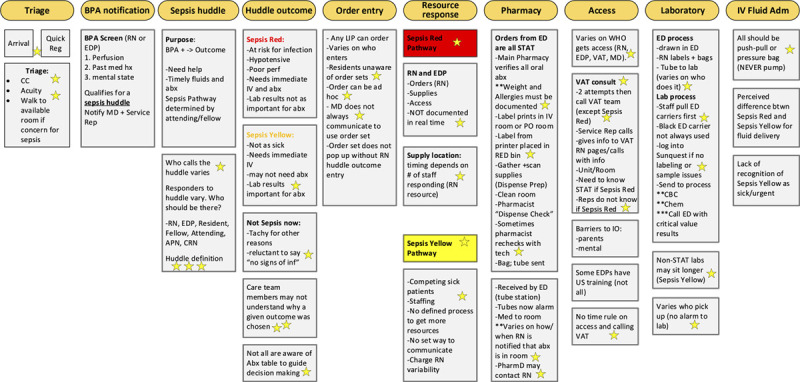
Kaizen process map. BPA, best-practice alert; CBC, complete blood count; CC, chief complaint; EDP, emergency department paramedic; IO, interosseous; MD, Medical Doctor; RN, registered nurse; VAT, vascular access team.

The event focused on a facilitated process mapping activity where participants described the sequential steps in the care of a patient with suspected sepsis based on vignettes (Fig. 2). Facilitators prompted and monitored discussions between the ED and colleagues from the pharmacy, laboratory, and vascular access teams to ensure a clear understanding of contingent workflows impacting sepsis care.

The process map began with a patient’s presentation to the ED and included triage, rooming, sepsis huddle procedures, and treatment workflows. The exercise concluded with the time of initial IV fluid bolus delivery and antibiotic administration. Each step was documented on a large poster for full visibility by all participants. Alternate flow diagrams indicated variations in workflows and were marked as discussion points. Facilitators emphasized describing processes as accurately as possible and dissuaded tendencies to focus on “ideal” state perceptions. Following this exercise, a gap analysis informed barrier categorization, and participants used a prioritization matrix to sort emerging solutions.

## RESULTS

**Fig. 3.**
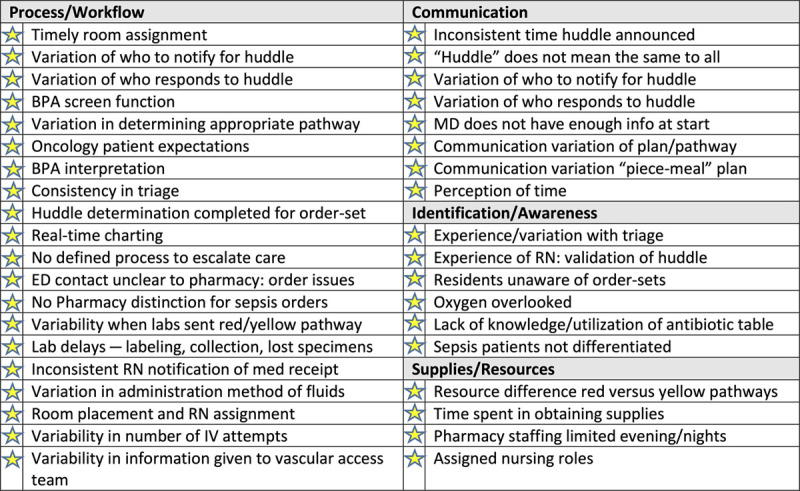
Kaizen gap analysis. BPA, best-practice alert.

Thirty-seven staff members across 17 disciplines participated in the event. Simultaneous identification of gaps during the process mapping highlighted opportunities for improvement in ED sepsis care processes and procedures (Fig. 3).

**Fig. 4.**
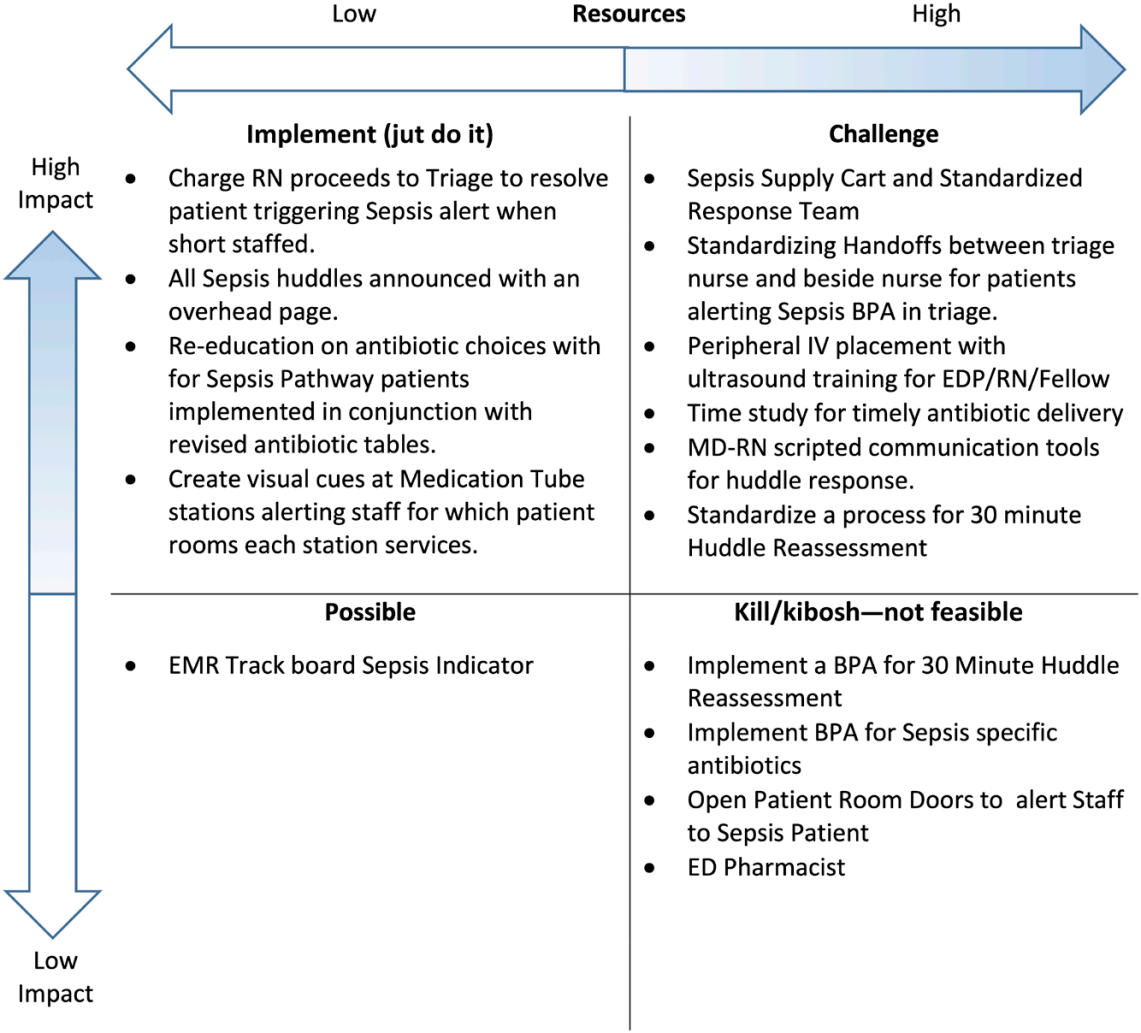
Kaizen PICK chart. BPA, best-practice alert; EDP, emergency department paramedic; MD, Medical Doctor; RN, registered nurse.

A prioritization matrix, which demonstrates the impact on the *y* axis and effort on the *x* axis, addressed the identified gaps and aided in our next steps. A PICK chart facilitated the visualization of high-yield interventions (Fig. 4). The acronym comes from the labels of each quadrant: Possible (little effort and low impact), Implement (little effort and high impact), Challenge (high effort and high impact), Kill/Kibosh (high effort and low impact).^13,14^

**Fig. 5.**
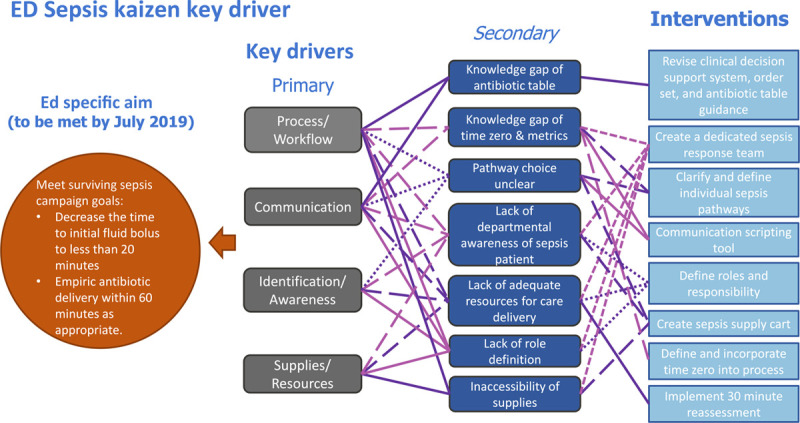
Kaizen key driver diagram.

After the Kaizen, team leaders reviewed the process map, gap analysis, and PICK chart and organized a key driver diagram to guide improvement activities. Based on these findings, the team identified 4 key drivers: process/workflow, communication, identification/awareness, and resource/supplies (Fig. 5).

### Process/Workflow

Process and workflow issues affected the timely delivery of care in all identified barriers.

Patient triage, based on room availability at our facility, led to two distinct workflows where the vital sign-based electronic trigger could fire. As a result, the “time zero” location was variable, leading to delays in patient care and treatment. Additional delays in treatment were attributed to inaccurate order entry and failure to use the sepsis order set bundles.

### Communication

Communication and education gaps among the care team played a prominent role in delayed recognition and treatment of sepsis patients. Lack of clear communication between nursing staff and providers surfaced as a central theme throughout. Providers identified a need for additional information to understand why a huddle initiation occurred, causing delays and miscommunication in pathway determination. Nurses identified that providers did not always clearly state which pathway they chose or why.

### Identification/Awareness

When a sepsis patient was being treated in the department, lack of awareness was identified as a contributor to delayed treatment response. Additionally, a lack of shared understanding of fluid resuscitation goals within the sepsis pathway led to a delayed sense of urgency and insufficient resource allocation for a subset of patients. Delays surrounding antibiotic administration were multifactorial. Lack of awareness of the hospital-wide antibiotic guidance table, included within the pathway, led to unnecessary calls to consulting services. Concerns for antibiotic stewardship led to delays in ordering. Additionally, remote pharmacy location and lack of awareness of antibiotic arrival to the ED via a pneumatic tube system further delayed administration.

### Supplies/Resources

Staff noted barriers to timely access to sepsis supplies and an inability to adequately leverage available staff to care for patients with sepsis concerns. Poorly defined roles and responsibilities of the care team members, nonspecific resource allocation, and lack of visual cues of a rapid resource response contributed to care delays. In the pediatric population, particularly in the setting of suspected sepsis, vascular access is often challenging and may require escalation of resources and use of the hospital’s vascular access team. Unclear messaging to this team led to additional care delays. Finally, the ED’s geographic layout, along with competing priorities in a high-volume/high acuity setting, contributed to barriers in timely treatment.

Using the gap analysis, PICK chart, and key drivers, stakeholders designed comprehensive interventions focused on standardizing sepsis treatment. Solutions included creating a sepsis response team with defined roles and responsibilities and the use of an overhead huddle call to bring team members to the bedside. A scripting communication tool was used for patient care hand-offs and pathway initiation. It improved the use of existing order sets previously developed to reduce delays and variations in care. A 30-minute reassessment by the provider was added to provide additional clarity to clinical decisions and encourage improved team dynamics. The group suggested a dedicated and brightly colored sepsis response cart to rapidly access necessary supplies and serve as a visual cue for departmental awareness. The creation of a sepsis response team assured additional resource support would be available.

**Fig. 6.**
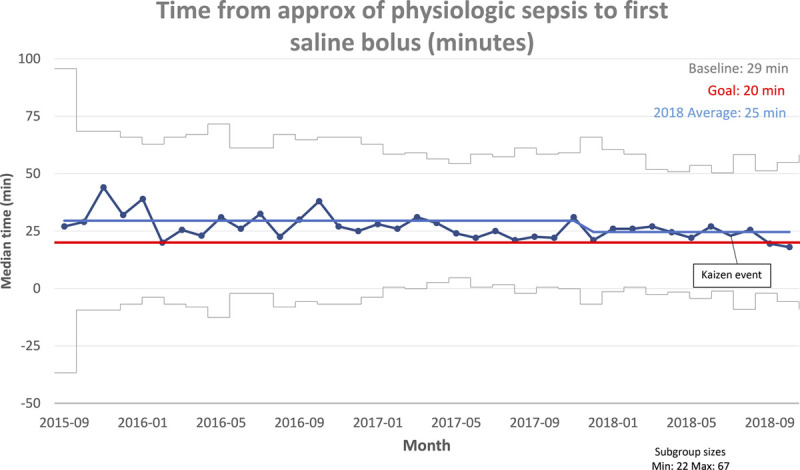
Statistical process control chart.

The availability of both supplies and additional team members (resource members) critical to the care of patients with severe sepsis was paramount to the initiative’s success. Following the Kaizen event, a reduction in initial fluid administration time was demonstrated before the formal implementation of prioritized interventions (Fig. 6). This initial, immediate improvement was attributed to frontline participation and engagement in solution generation.

## DISCUSSION

Although the Kaizen methodology is not a novel approach in healthcare, this event was the first large-scale multidisciplinary improvement exercise related to the quality of sepsis management in our institution. It can serve as a case study for EDs with emerging QI programs. Our ED sepsis team considered this event a success for many reasons. First, our ED’s feasibility to employ Kaizen events for QI initiatives was proven. This event yielded a high level of engagement among senior leadership, who prioritized time for over 30 multidisciplinary participants to attend. Staff’s feedback following the event was mostly positive, with requests to attend future improvement Kaizens. Additionally, this event yielded actionable ideas to create a pilotable intervention to further our progress towards achieving the Surviving Sepsis Campaign goals.^15,16^

This rapid improvement event provided frontline staff the opportunity to create a shared understanding of our institution’s current sepsis care delivery processes. The process mapping exercise highlighted multiple areas of care team variability and workflow barriers to timely treatment. Following the event, our observation of frontline staff’s real-time engagement in improving sepsis care during clinical shifts was the most valuable outcome. This result suggests that Kaizen is instrumental in changing culture and can serve as a meaningful model to sustain frontline staff’s involvement in future improvement efforts.

Previous sepsis QI studies have included barrier assessments to facilitate the implementation of sepsis care interventions.^17–23^ The barriers identified in our rapid improvement event are similar to those identified in other pediatric EDs. First, determining and understanding when to assign “time-zero” remains a controversial aspect of pediatric sepsis care.^18^ Early recognition of sepsis itself represents a significant barrier and has been the subject of most interventional studies to date.^1,6,19,24–26^ As part of one of these initiatives, Cruz^27^ conducted a root-cause analysis of barriers to sepsis care, identifying a lack of standardization of empiric antibiotics and diagnostic tests and a lack of medication prioritization as significant barriers. Although formalized protocols and guidance for antibiotics were in place before this exercise, the universal use of sepsis order bundles and empiric antibiotic guidance was lacking. In another interventional study, Paul et al^17^ identified knowledge gaps, vascular access, and poor communication among the clinical team as barriers to timely sepsis care.^28^ The root cause of poor communication in this study differed from our own experience. In this study, communication gaps were due to staff familiarity and trainee transition. In contrast, our gaps were based on the unclear articulation of sepsis care and pathway concerns between nursing and physician teams.

Our rapid improvement event underscores the need to understand the local context to generate solutions and buy-in relevant and endorsed by stakeholders. Assessing local barriers via a multidisciplinary perspective is a recognized approach to enhancing the successful implementation of QI interventions.^18,29^ This event further demonstrated the benefit of revisiting collaborative, multidisciplinary engagement strategies, and QI processes necessary to build a culture of continuous improvement and understand emerging barriers that may impede the success of long-standing QI initiatives.^30^

The Kaizen concluded with a focused plan to begin iterative improvement cycles within 3 months of proposed interventions: a unit-based sepsis response team with defined roles and responsibilities, a standardized communication tool, a sepsis supply cart delivered to the bedside, and a 30-minute bedside reassessment for all presumed sepsis patients. The literature supports the use of allocated resources for sepsis response teams.^22^ In other studies, these teams demonstrated improvement in patient outcomes in the critical care services and EDs.^31,32^ Scripted communication tools used by these teams have also led to improved provider-to-provider communication.^33^ Solutions chosen for implementation following this event addressed several significant gaps and were engineered to achieve a high impact in overall sepsis care delivery. Finally, these interventions will be tested to evaluate if initial gains in time to fluid administration immediately following the Kaizen can be further supported and sustained.

## LIMITATIONS

We acknowledge several limitations of the event specifically and Kaizen methodology generally. First, Kaizen events of this magnitude benefit from the reservation of multiple days to adequately address all barriers and determine possible solutions for further interventions.^34^ Our limited timeframe of 6 hours prevented a full assessment of this complex problem and will require future efforts to continue improvements; however, this is a recognized challenge in health care QI.^29^ The absence of representation by resident trainees for this event may have contributed to gaps in knowledge of other inefficient processes unknown to the participants involved. The Kaizen was held in July, an optimal time to hold QI events as ED patient volumes are typically low. However, this timeframe did not easily allow for trainee participation. Although we made attempts to reach this group, this was a one-time multidisciplinary exercise and the team understood that not all desired participants could be in attendance.

Rapid improvement events are expensive, mainly due to costs related to staff participation and room reservation.^35^ For our event, staff attendance approximated 280 staffing hours. The facilitator’s time to prepare the event totaled another 80 hours. Knowing this limitation, we planned our Kaizen strategically to be held during a low-volume time as not to accrue overtime pay for attendees. We incurred no room rental fees. Additionally, we chose to pilot the Kaizen methodology using a team trained in QI methodology and aligned our goals with institutional priorities to generate additional buy-in from those in attendance. Future efforts to implement the Kaizen methodology for QI should consider the availability of these resources and the projected costs and an assessment of gains realized directly through the QI efforts.

## CONCLUSIONS AND NEXT STEPS

Taking the time and designing an event that allowed frontline care team members to openly state variability and barriers to sepsis care delivery proved invaluable to identify improvement plan-do-study-act cycles. Frontline staff involvement in root cause barrier identification and solution generation uncovered opportunities for improvement that would otherwise have gone unnoticed. Based on this event’s success, the ED plans to incorporate rapid improvement events into future QI projects. Kaizen enabled us to improve processes and sustain momentum for continuous improvement, which is needed to engrain QI in practice.

## DISCLOSURE

The authors have no financial interest to declare in relation to the content of this article.

## ACKNOWLEDGMENTS

We acknowledge the time, participation, and candid discussion of all frontline providers who attended the rapid improvement event. We appreciate the foundational support of departmental leaders to achieve frontline provider attendance. Finally, we acknowledge those involved in building the structural platform surrounding the care of sepsis patients in the ED, Lurie Children’s Center for Excellence team members, and members of the data analytics and reporting team. The authors gratefully acknowledge the Children’s Hospital Association Improving Pediatric Sepsis Outcomes Collaborative, a multihospital, multiyear collaborative that provides centralized, nonmonetary resources to support pediatric sepsis QI work.

## Supplementary Material


