# Supplementary figures and images for: Multidisciplinary Kaizen Event to Improve Adherence to a Sepsis Clinical Care Guideline
Source: Pediatr Qual Saf. 2021 Jun 23;6(4):e435. doi: 10.1097/pq9.0000000000000435 (PMC8225368; doi:10.1097/pq9.0000000000000435)

Supplemental Figure 1

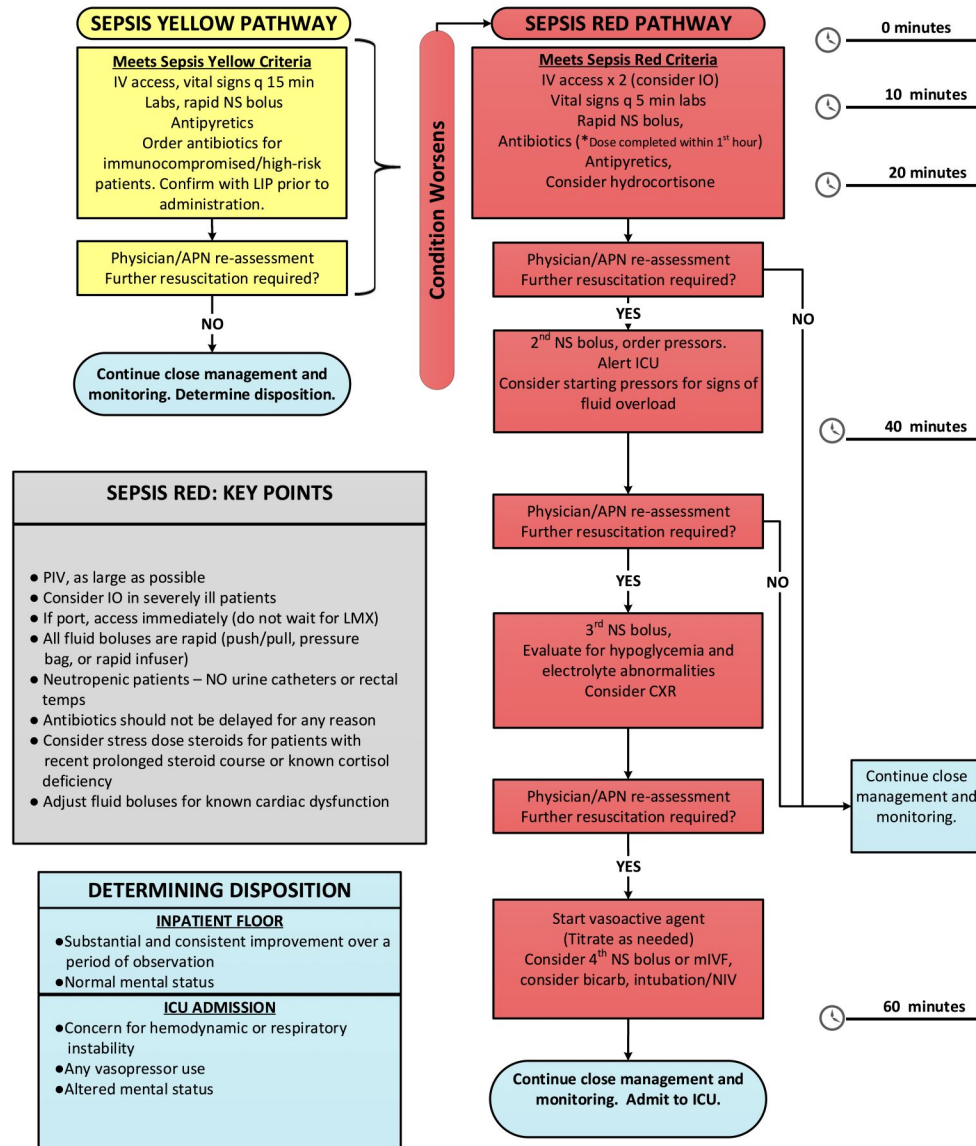

Supplement: Supplementary file 1 [file pqs-6-e435-s001.pdf]
